# The Treatment of Indolent Ulcers by Means of Sheet Lead

**Published:** 1879-09

**Authors:** 


The Treatment of Indolent ulcers by Means of Sheet Lead.—A good deal of attention has been attracted during the past year to the American treatment of indolent ulcers by means of Dr. Martin's India rubber bandages, and the reports received on all sides as to the value of this method are eminently satisfactory. I would, however, urgently request that a trial be given to the system which I was in the habit of adopting in all such cases at St. Bartholomew's Hospital, Chatham, some thirteen years ago, viz.: the application of sheet lead, molded to the shape of the leg and kept on by an ordinary calico bandage. The size of the lead should be sufficient to cover the ulcer completely, and lap it a little over the whole skin ; the edges and angles should be well rounded, so as not to chafe or irritate ; it should be about an eighth of an inch in thickness, and moulded very accurately to the shape of the leg, so as to allow of no indent being apparent on the surface. After it has been carefully fittted the leg should be bandaged from the toes upward, and all that then need to be done is to uncover the ulcer night and morning and allow some water from a spongue to trickle over it. The granulations should never be touched with the spongue itself. I believe that the rationale of this treatment is pressure, the same as in the case of the elastic bandage, though there may be also some action produced by the secretions upon the lead, as is said to take place when the lead nipple-shields are used. The great advantages of the system proposed are simpicity and cheapness, though, as regards the former, I think it must yield the palm to the elastic bandage. It would appear that in some parts of Africa the natives use sheet copper, and with some success, but I cannot say I have ever tried it myself.—*London Practioner.*

